# Statistical test for detecting community structure in real-valued edge-weighted graphs

**DOI:** 10.1371/journal.pone.0194079

**Published:** 2018-03-20

**Authors:** Tomoki Tokuda

**Affiliations:** Okinawa Institute of Science and Technology Graduate University, 1919-1, Tancha, Onna-son, Okinawa, Japan; Universitat Rovira i Virgili, SPAIN

## Abstract

We propose a novel method to test the existence of community structure in undirected, real-valued, edge-weighted graphs. The method is based on the asymptotic behavior of extreme eigenvalues of a real symmetric edge-weight matrix. We provide a theoretical foundation for this method and report on its performance using synthetic and real data, suggesting that this new method outperforms other state-of-the-art methods.

## Introduction

Clustering objects based on their similarities is a basic data mining approach in statistical analysis. In particular, graphical data (or network data) that reflect relationships between nodes, are often acquired in various scientific domains such as protein-protein interaction, neural networks, and social networks [1], which potentially provide useful information on the underlying structure of the system in question.

**Fig 1:**
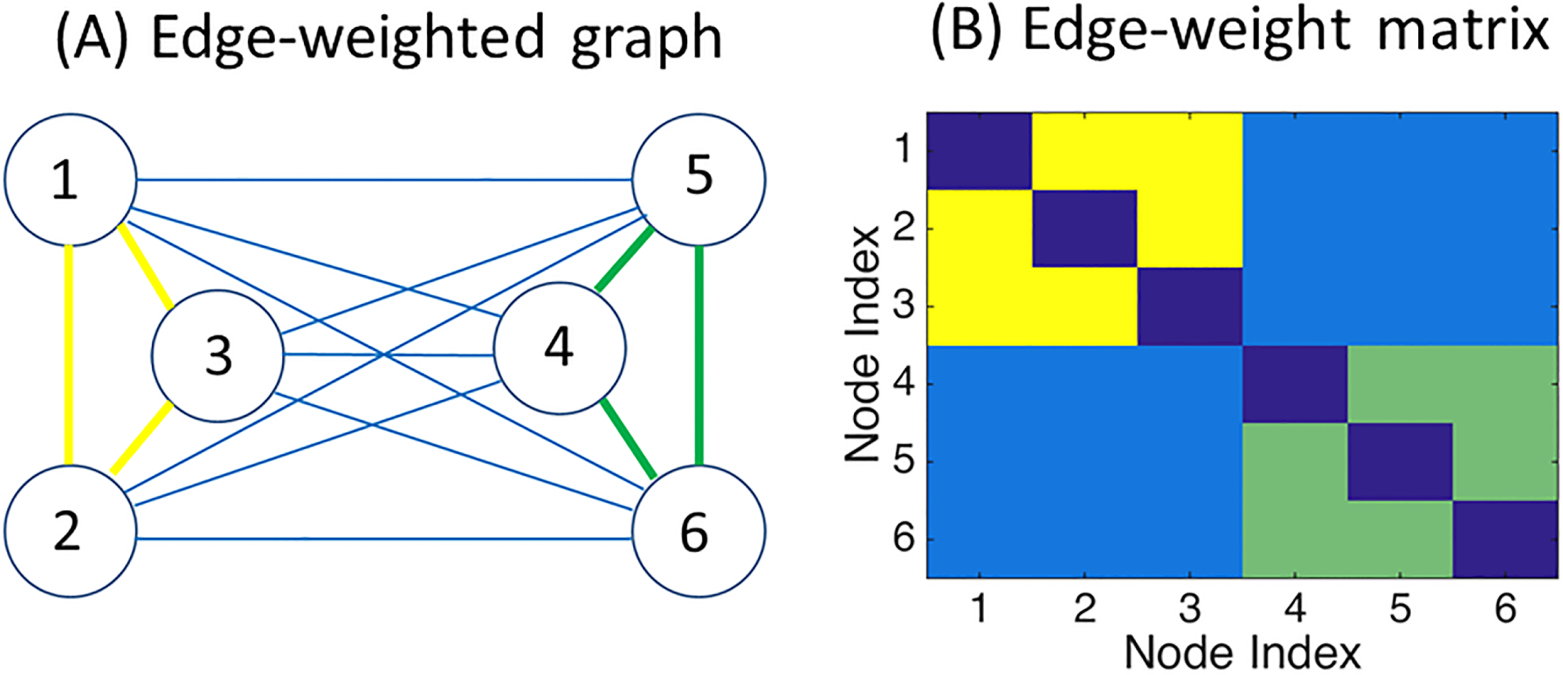
Illustration of two-way community structure in a graph. Panel (A): Graphical representation (edge-weighted graph). Panel (B): Matrix representation (edge-weight matrix), where strengths of relationships between nodes are denoted in color.

Specifically, our interest is to detect possible ‘community’, or cluster structure of undirected graphs, which is defined as block structure of a graph (Fig 1A), where the corresponding edge-weight matrix consists of several cluster blocks (four cluster blocks in Fig 1B). To detect such structure, a number of clustering methods have been proposed in the statistical physics and information theory literature [2–4]. Mainly, there are four approaches: graph partitioning, hierarchical clustering, partitional clustering, and spectral clustering [1, 4, 5].

However, the conventional framework for analysis of community structure is typically an unsigned graph in which an edge weight is constrained to be non-negative. Recently, increased attention has been paid to analyzing signed graphs that allow negative weights [6]. Indeed, in real data, it is often essential to account for negative, as well as positive relationships, for a better understanding of the underlying community structure in a graph such as a social network. Most methods in the literature, however, address this problem in a rather limited framework in which edge weights within a cluster are positive while those between clusters are negative (i.e., weakly balanced structure) [6]. On the other hand, how to cluster nodes in a more general framework, such as negative edge weights within a cluster, remains an open question [7].

In the present paper, we address the question of community detection in a real-valued graph. Let us consider a general framework for community structure as follows. We assume that edge weights are independently generated from a generative model that is specific to a particular cluster block, which characterizes a distribution of edges in each cluster block. Further, we assume that these distributions are distinguishable in terms of their mean and variance. For this framework, as a first step toward addressing a clustering problem, we aim to develop a statistical method for testing the existence of underlying community structure.

From the theoretical point of view, there is the issue of detectability of community structure. In the case of unweighted graphs, this issue has been intensively studied because of both mathematical and physical interest [8–10]. In the situation in which an edge connection is generated by a probability *P*_*ab*_ = *c*_*a*,*b*_/*n* where *c*_*a*,*b*_ is constant and *n* is the number of nodes, it has been shown that it is impossible for any algorithm to detect underlying community structure (as *n* → ∞) under certain circumstances. Further, it is shown that instead of a conventional adjacency matrix, a non-backtracking matrix, which represents non-backtracking walks in a network, provides a better platform for detection algorithms [11]. In the present paper, however, we focus on the case in which a generative model for edges has fixed parameters, irrespective of the number of nodes *n*. In the context of unweighted graphs, this suggests that *P*_*a*,*b*_ = *c*_*a*,*b*_. In this situation, it was shown that it is possible to detect community structures (in case of bisection) as *n* goes to ∞ [12, 13]. In the present paper, we consider such a case.

Regarding statistical tests on community structure, several methods have been proposed in the context of unsigned (weighted or unweighted) graphs [1]. A common approach to this problem is to evaluate the stability of cluster solutions when the data in question are noisy [14, 15]. If similar cluster solutions are obtained for graphs with some perturbation of edge-weights, this suggests the stability of the cluster solution for the original graph, providing the evidence of the community structure. The bootstrap method employs [16] a similar approach. A second approach is based on comparisons of cluster solutions for the original graphs with solutions of randomly permuted graphs. As a statistic for testing significance, the entropy of graph configurations [17], or ‘C-score’ focusing on the lowest internal degrees [18] have been proposed. The common feature of these state-of-the-art methods is that a cluster solution to a given graph is required for testing. In other words, the test result depends on the clustering method employed. In this sense, these methods test the significance of a resulting cluster solution, rather than the existence of community structure itself. For the general framework of our interest, such an approach is not applicable because appropriate clustering methods are not readily available. In [19–21], the spectral homophily of a multi-type random network has been proposed to capture connectivity between communities. This method uses the second largest eigenvalue of a symmetric matrix with expected fractions of the links where the partition in communities is exogenous. However, it is not straightforward to apply their results to our setting of real-valued edge-weights. Moreover, we consider a situation in which the partition in communities is not exogenous.

We propose a general method for testing community structure of edge-weighted graphs with real-valued weights, which does not require a cluster solution. Our method is based on the asymptotic behavior of eigenvalues of the normalized weight matrix of graph, which is described by Wigner semicircular law when there is no community structure. As in our approach, in the case of binary-valued graphs, a statistical test for community structure has recently been proposed [22], based on the exact asymptotic behavior of (maximum) eigenvalues. However, that method is not directly applicable to real-valued graphs that account for both mean and variance, because the Bernoulli distribution assumed in their method cannot properly capture these quantities. Our method provides a nontrivial extension of community structure detection to real-valued graphs, and broad applications to network data. In the following sections, first, a theoretical foundation for our method is provided. Second, it is shown that our method outperforms other methods with synthetic data. Third, we apply our method to real data.

## Method

Our statistical test on community structure is based on the probability distribution of eigenvalues of the normalized edge-weighted matrix (we define ‘normalization’ later). We make the best use of asymptotic results on such a distribution when there is no community structure, which has been intensively studied in the field of Random Matrix Theory of Theoretical Physics [23]. In this section, we provide a theoretical foundation for our statistical test.

### Setting

**Fig 2:**
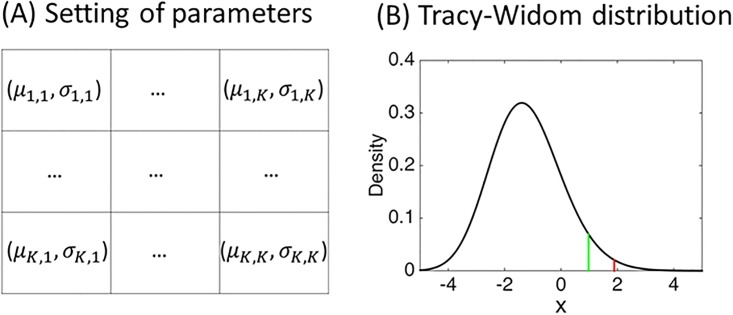
Setting of community structure and Tracy-Widom distribution. Panel (A): Illustration of the setting of community structure in a matrix representation where nodes are arranged in the order of cluster labels. Each cluster block is characterized by mean *μ* and standard deviation *σ* with cluster block index (*k*, *k*′). Panel (B): The density function of the Tracy-Widom distribution for Gaussian orthogonal ensembles with *β* = 1 (the first derivative of *F*_1_(*x*) in Eq (7)), generated by the function *dtw* in R-package {RMTstat}. The critical values at significance level *α* = 0.05 and *α*/4 = 0.0125 are 0.979 (green line) and 1.889 (red line), respectively.

We consider a clustering problem of nodes for undirected edge-weighted graphs *G* = (*V*, *E*) where *V* consists of *n* vertices {*v*_1_, …, *v*_*n*_}, and *E* is represented by the edge-weight matrix ***W***_*n*_, which is a *n* × *n* symmetric (real Hermitian) matrix with elements $w_{i,j}=w_{j,i}\in \mathbb{R}$ and *w*_*i*,*i*_ = 0 (ℝ denotes a set of real numbers). Let us assume that there are *K* clusters of nodes, denoting them as *c*_1_, …, *c*_*K*_. We define a cluster block (*k*, *k*′) as a set of weights *w*_*i*,*j*_ such that nodes *i* and *j* belong to the cluster *c*_*k*_ and *c*_*k*′_, respectively: *v*_*i*_ ∈ *c*_*k*_ and *v*_*j*_ ∈ *c*_*k*′_ (1 ≤ *k*, *k*′ ≤ *K*). Here, we assume that each off-diagonal weight *w*_*i*,*j*_ is independently drawn from a certain distribution. With this assumption, we define a *K*-way community structure as characterized by different distributions in *K* × *K* cluster blocks. To elaborate this definition, we assume the following distribution for each cluster block:

$$\begin{array}{ccc} w_{i,j} & ∼ & g_{k,k^{\prime }}\;\;\left(i\neq j\right)\\ g_{k,k^{\prime }} & = & \mu_{k,k^{\prime }}+g\times \sigma_{k,k^{\prime }}, \end{array}$$ (1)

where *v*_*i*_ ∈ *c*_*k*_, *v*_*j*_ ∈ *c*_*k*′_, and *g* is a certain probability distribution. This definition suggests that a pair of parameters (*μ*_*k*,*k*′_, *σ*_*k*,*k*′_) characterizes each cluster block, hence, community structure (Fig 2A). Note that in this definition we exclude the degenerate case in which *μ*_*k*,*k*′_ = *constant* and *σ*_*k*,*k*′_ = 0 such that variances become zero for the whole set of {*w*_*i*,*j*_}.

Since the community structure of interest is based on differences of weight distributions, it is translation- and scale-invariant for all weights. Hence, to simplify the problem, as a preprocess, we standardize off-diagonal elements of ***W***_*n*_ using all off-diagonal weights *w*_*i*,*j*_(*i* ≠ *j*) so that the mean is zero and the variance one. We denote as *S* the mapping that standardizes the edge-weight matrix in this way, transforming each element of the matrix as

$$\begin{array}{ccc} S: & & w_{i,j}\to \left(w_{i,j}-\mu \right)/\sigma \;\;\text{for}\;i\neq j\\ & & w_{i,i}\to 0, \end{array}$$ (2)

where *μ* and *σ* are the mean and the standard deviation of the whole off-diagonal elements {*w*_*i*,*j*_}. Practically, these mean and standard deviation may be replaced by the empirical counterparts *μ*_*emp*_ and *σ*_*emp*_. For the standardized edge-weight matrix *S*(***W***_*n*_), we assume that the mean and the standard deviation of *g* in Eq (1) are zero and one, respectively. In this setting, the mean and the standard deviation in cluster block (*k*, *k*′) are *μ*_*k*,*k*′_ and *σ*_*k*,*k*′_, respectively. The differences of these parameters distinguish between clusters in terms of the first and second moments, while controlling higher moments than two. Using this setting of community structure, we define no community case as a single community with *K* = 1 where *μ*_*k*,*k*′_ = 0 and *σ*_*k*,*k*′_ = 1 for *S*(***W***_*n*_). Note that since *g* is arbitrary, including a mixture distribution of a certain distribution family, our definition of no community structure includes the case in which each weight is generated from a specific distribution in a list of distributions in random order. Importantly, when we shuffle the off-diagonal elements ***W***_*n*_ at random (in element-wise manner), the community structure always disappears. Indeed, in such a case, each element $w_{i,j}^{\prime }$ of the shuffled matrix $W_{n}^{\prime }$ independently and identically follows the mixture distribution consisting of different components, i.e., ∑_*k*,*k*′_ *π*_*k*,*k*′_ *g*_*k*,*k*′_ where *π*_*k*,*k*′_ is the proportion of elements of cluster block (*k*, *k*′) for the original matrix ***W***_*n*_. We use this property for our statistical test as an alternative way to estimate confidence intervals.

### Statistical test

In this section, we develop a statistical test for the existence of community structure defined in the previous section (i.e., *K* = 1 vs. *K* > 1). We base our test on the asymptotic behavior of the eigenvalues of *S*(***W***_*n*_) (*n* goes to ∞) when there is no community structure. A useful result of Random Matrix Theory in this context is that if the elements of an infinite dimensional symmetric matrix ***X*** independently follow a certain distribution with mean zero and variance one, then the empirical (random) distribution of the eigenvalue *λ* of $X_{n}/\sqrt{n}$, where ***X*_*n*_** is the principal submatrix of ***X*** for the first *n* rows and columns, converges almost surely to a Wigner semicircular distribution as *n* goes to ∞ (semicircular law).

$$\begin{array}{c} f_{sc}\left(\lambda \right)\equiv \frac{1}{2\pi }\sqrt{4-\lambda^{2}}. \end{array}$$

Note that this law holds for any generative distribution of the elements in matrix ***X*** (as long as independently drawn), which is referred to as the universality property of the law. Also, this law holds even if we replace diagonal elements with zero’s, as in our case. Further, strong Bai-Yin theorem suggests that with the additional condition of the distribution of each element (namely, the existence of a fourth moment), the largest magnitude of eigenvalues is almost certainly bounded by 2. These two theorems imply that the largest magnitude of eigenvalues almost surely converges to two ([24], p.136).

In order to apply this property to our context, we consider a normalization mapping of edge-weight matrix ***W***_*n*_, transforming each element of the matrix as

$$\begin{array}{ccc} T: & & w_{i,j}\to S\left(w_{i,j}\right)/\sqrt{n}, \end{array}$$ (3)

where *S* is the standardization mapping in Eq (2). Now, let us assume that the elements in an edge-weight matrix ***W***_*n*_ are generated as in Eq (1). In this setting, if the largest magnitude of eigenvalues of *T*(***W***_*n*_) does not converge to two, then, there should be some *K*-way community structure in the graph (*K* > 1) because of our assumption in Eq (1) (Note that without the assumption in Eq (1), this property does not hold. For instance, one can make a scale-free graph where the eigenvalues do not follow the semicircular law [25]). However, the converse argument does not necessarily hold. That is to say, the fact that the convergence of the largest magnitude of eigenvalues to two does not imply that there is no community structure (i.e., *K* = 1). A simple counter example is given as follows (proof in S1 Appendix).

**Example 1.** *Let **W**_n_ be a n* × *n symmetric edge-weight matrix that has K-way community structure with the same cluster size (n/K) as defined in the previous section. Suppose that μ_k,k′_ = 0 for* ∀*k, k′*, $\sigma_{k,k^{\prime }}^{2}=0$ *for k* ≠ *k′, and* $\sigma_{k,k}^{2}=1$. *Then, the largest magnitude of eigenvalues of T(**W**_n_) almost surely converges to two as n goes to* ∞.

Nonetheless, in our setting, we can show that an additional condition on the eigenvalue distribution for an exponentially mapped edge-weight matrix ensures that the converse argument also holds. For this purpose, we introduce the exponential mapping *Exp* that transforms each element of ***W***_*n*_ as

$$\begin{array}{ccc} Exp: & & w_{i,j}\to \exp\left(t\times w_{i,j}\right)\;\;\text{for}\;i\neq j\\ & & w_{i,i}\to 0, \end{array}$$ (4)

where t∈ℝ is a tuning parameter (we do not explicitly denote the dependence of *Exp* on *t* because of cluttering). Subsequently, we define the normalization mapping *T*_*e*_ for the exponentially transformed matrix as

$$\begin{array}{ccc} T_{e}: & & w_{i,j}\to S\left(Exp\left(w_{i,j}\right)\right)/\sqrt{n}. \end{array}$$ (5)

Now, the following theorem provides a necessary and sufficient condition for the existence of community structure (proof in S2 Appendix).

**Theorem 1.** *Let **W**_n_ be a n* × *n weight matrix defined in the previous section with the fixed proportion of cluster sizes* (*r*_1_, …, *r_K_*) *and the pairs of fixed parameters* {(*μ_k,k′_, σ_k,k′_*)}(*k, k′* = 1, …, *K*). *Suppose that there exists the moment-generating function M*(*t*) *in an open interval containing zero for g (g is defined in* Eq (1)). *Then, the following statements (C1) and (C2) are equivalent*:

*(C1) There is no community structure (i.e., K* = 1*)*
*(C2) Each of the largest magnitudes of eigenvalues of T*(***W***_*n*_) *and T*_*e*_(***W***_*n*_) *for any non-zero real value t*_0_ ≠ 0 *almost surely converges to two, as n goes to* ∞.

Theorem 1 motivates us to use the largest magnitude of eigenvalues of edge-weight matrix to establish a statistical test on the null hypothesis *H*_0_:

$$\begin{array}{c} H_{0}:\text{There}\;\text{is}\;\text{no}\;\text{community}\;\text{structure}. \end{array}$$ (6)

Practically, to test the null hypothesis *H*_0_, we focus on positive and negative extreme values of eigenvalues. The largest eigenvalue may deviate positively from two, while the smallest eigenvalue may deviated negatively from -2.

#### Comments

**Fig 3:**
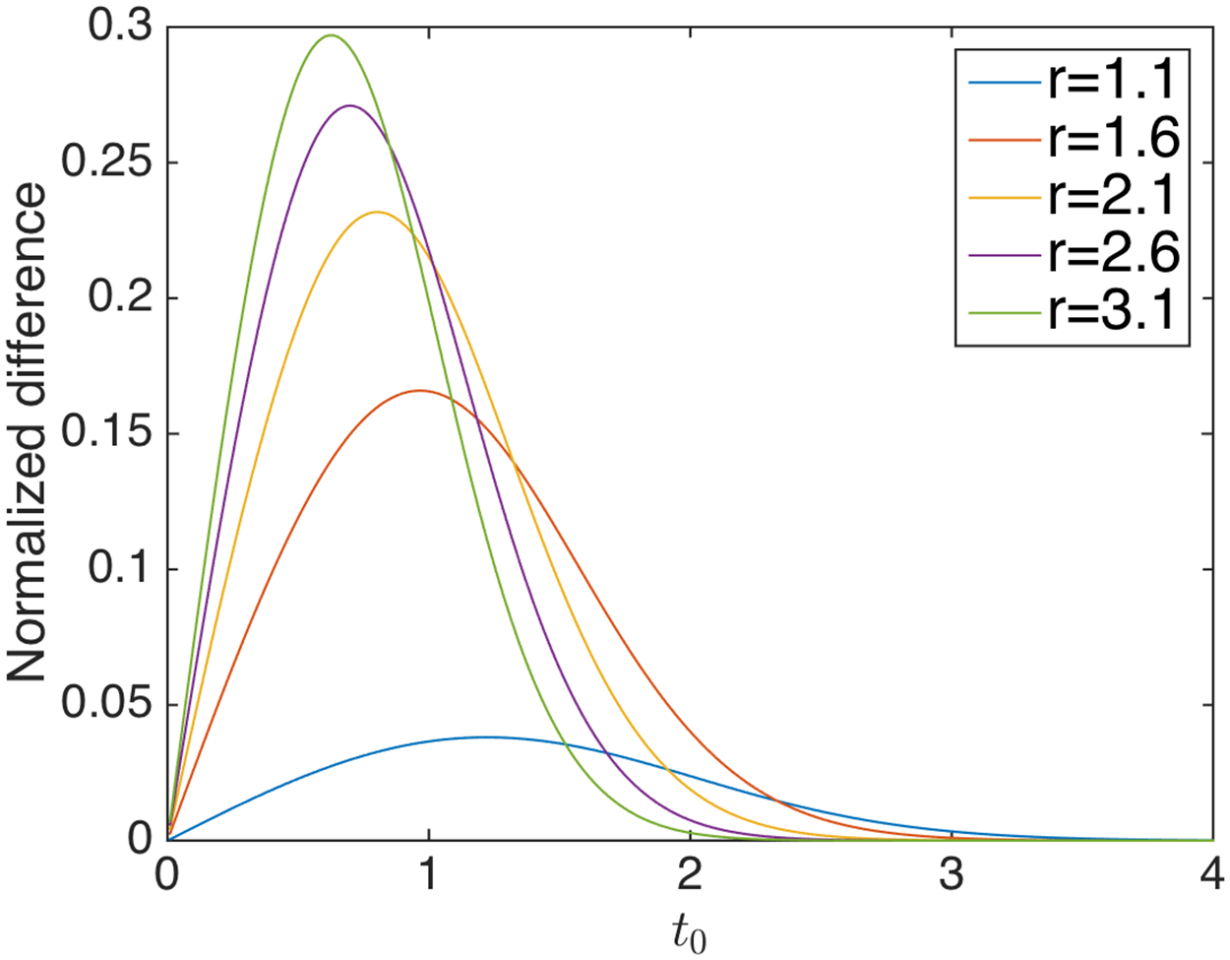
Normalized differences of exponentially transformed variables between normal distributions *N*(*μ*, *σ*_1_) and *N*(*μ*, *σ*_2_). The X-axis denotes *t*_0_ in Theorem 1 and the Y-axis normalized difference. We set $r=\sigma_{1}^{2}/\sigma_{2}^{2}$ to 1.1, 1.6, 2.1, 2.6, and 3.1.

1. Strictly speaking, the independent assumption on weights is broken if we transform them by *T* or *T*_*e*_ using an empirical mean and standard deviation *μ*_*emp*_ and *σ*_*emp*_. For simplicity, however, we ignore such an effect in the present paper.
2. Our method is not applicable to a directed graph, because in that case an edge-weight matrix becomes non-symmetric; hence, Theorem 1, which is based on properties of eigenvalues of symmetric matrices, does not hold.
3. The spectral method in [19–21] takes a slightly different approach from our method. In the context of unweighted graph, they consider a symmetric matrix representing an expected fraction of edges between two communities (hence, the summation of entries in a row is one). Further, the size of the matrix in their approach is *K* × *K* while that of our method is *n* × *n*. Moreover, in their approach, the second largest eigenvalue is considered because the largest eigenvalue is constant (always one). As other community detection methods, it is not trivial to generalize their method to real-valued graphs.
4. Theorem 1 implies that if there is no community structure (i.e., edges are i.i.d. generated), the ratio of the second largest eigenvalue to the largest eigenvalue converges to one. This is a general property to a symmetric matrix. This result is contrasted with Brody’s conjecture that for a positive non-symmetric matrix in which all edges are i.i.d. generated, the ratio of the second largest eigenvalue to the largest eigenvalue goes to zero [26–28].
5. The exponentially transformed matrix in Theorem 1 does not replace non-backtracking matrix in [11]. Rather, the exponentially transformed matrix serves to capture differences of variances in generative models.
6. In the current setting, means and variances in generative models are fixed. One may wonder how much this condition can be relaxed. From the argument about unweighted graphs by [12, 29], we speculate that one may be able to relax the condition such that the difference of means may be larger than Ω(log *n*/*n*). Indeed, assuming that this condition holds after standardizing the edge-weight matrix, Eq (3) in S2 Appendix becomes λ_1_(*M*_*n*_) ≥ *A*(log *n*)^2^. Hence, in this relaxed condition as well as in non-relaxed condition, it holds that if there is community structure with equal variances the largest magnitude of eigenvalues does not converge to two (the first part of the proof of Theorem 1). Moreover, using the relaxed condition, it can be shown that for the unstandardized matrix, mean differences should be also larger than Ω(log *n*/*n*). Note that from the prime number theorem [30], the reciprocal of log *n*/*n* denotes the number of prime numbers less than *n*. This observation provides us the following interpretation of the results. If we assume that the community size is the same across different communities, the number of prime numbers denotes the number of irreducible topologies of communities (for example, four-community structure may be reduced to two-community structure by paring two communities while three-community structure is not reducible). We speculate that such an irreducible community structure is easier to detect than the remainder of structures and that if such structures are more available, the detection of community structure becomes easier. This interpretation suggests that the number of prime numbers may be inversely related to detectable differences of means, which is consistent with the lower bound Ω(log *n*/*n*).
7. One may wish to tune the value of *t*_0_ as follows. We consider two-community structure, assuming that edges within communities are generated by $N(\mu ,\sigma_{1}^{2})$ while edges between communities by $N(\mu ,\sigma_{2}^{2})$. The first moment (mean) of the exponentially transformed variable generated from *N*(*μ*, *σ*) is given by exp(*μt* + *σ*^2^*t*^2^/2) while the second moment is exp(2*μt* + 2*σ*^2^*t*^2^). Using these results, we can analytically evaluate the difference in means of the exponentially transformed variables between $N(\mu ,\sigma_{1}^{2})$ and $N(\mu ,\sigma_{2}^{2})$ normalized by the square root of the average of variances. It can be shown that in this case *μ* is irrelevant for the normalized difference. So, one may choose *t*_0_ that maximizes the normalized difference for a given *σ*_1_ and *σ*_2_, or, simply the ratio $r=\sigma_{1}^{2}/\sigma_{2}^{2}$. From Fig 3, one may choose *t*_0_ between 0.5 and 1.

The behavior of the largest eigenvalue has been well studied in the literature when elements of the edge-weight matrix ***W***_*n*_ are independently generated by certain symmetric distributions *g* (typically Gaussian, otherwise, its density function may be even with less heavier tails than Gaussian distributions) with mean zero and variance one for non-diagonal elements and with mean zero and variance two for diagonal elements. In this setting, the largest eigenvalue λ_*max*_ asymptotically follows the Tracy-Widom distribution for Gaussian orthogonal ensembles with parameter *β* = 1:

$$\begin{array}{c} \lim_{n\to \infty }P\left(\lambda_{max}\leq 2+x/n^{2/3}\right)=F_{1}\left(x\right), \end{array}$$ (7)

where $F_{1}(x)\equiv \text{exp}\{-(1/2)\int_{x}^{\infty }q(y)dy\}(F_{2}(x){)}^{1/2}$ with $F_{2}(x)\equiv \text{exp}\{-\int_{x}^{\infty }(y-x)q^{2}(y)dy\}$ where *q*(*x*) is the solution of Painlevé II equation *d*^2^*q*/*dx*^2^ = *xq* + 2*q*^3^ with the boundary condition *q*(*x*) ∼ Ai(*x*) as *x* → ∞ [31, 32]. Note that the Tracy-Widom distribution is for the maximum eigenvalue of a specific type of symmetric matrix (e.g., Gaussian ensembles) while the semicircular law holds for the distribution of eigenvalues in a general type of symmetric matrix (Wigner ensembles). Moreover, in our framework, the diagonal elements are all zero, which is a slightly different situation than the conventional assumption for the Tracy-Widom distribution. Nevertheless, because of the universality property of the Tracy-Widom distribution ([33], Theorem 21.4.3), we can safely apply Eq (7) to our context (obviously, our context satisfies the condition of universality that the diagonal part should be symmetric with a sub-Gaussian tail).

Using the Tracy-Widom distribution in Eq (7), we set confidence intervals for our statistical test as follows. For the normalized edge-weight matrix *T*(***W***_*n*_), we set the confidence interval *CI*_*max*_ of the largest eigenvalue λ_*max*_ at level *α*. Since the violation of the semicircular law occurs as the positive deviation from the expected value, we consider the one-sided confidence interval as (−∞, *q*) where *q* is a critical value at significant level *α*, i.e., *P*(λ_*max*_ ≥ *q*|*H*_0_) = *α*, which is estimated by *F*_1_(*x*) in Eq (7) (refer to the shape of its first derivative in Fig 2B). If the generative distribution *g* is not symmetric or is heavy-tailed, one may evaluate the distribution of the largest eigenvalues by means of a permutation test for *T*(***W***_*n*_). Though the permutation test may provide an accurate confidence interval, it is not computationally efficient because we need to compute eigenvalues a large number of times. Therefore, when the number of nodes is large, one may opt for the Tracy-Widom distribution to efficiently obtain confidence intervals. In addition to the largest eigenvalue, we also test the smallest eigenvalue λ_*min*_, which may violate the semicircular law (what matters is indeed the largest magnitude of eigenvalue). In this case, the confidence interval *CI*_*min*_ is given by (−*q*, ∞). In similar fashion, we test the largest and the smallest eigenvalue of the exponentially normalized weight matrix. We first standardize the data and then apply the mapping *T*_*e*_ where we set *t*_0_ to 1/2 as default. This results in the transformed matrix *T*_*e*_(*S*(***W***_*n*_)) (we denote the confidence intervals as $CI_{max}^{\prime }$ and $CI_{min}^{\prime }$, respectively). Since this procedure involves a series of four statistical tests, we set the level of significance to *α*/4 for each test, taking into account the Bonferroni correction (Algorithm 1; I(a) is an indicator function: 1 for correct *a*; 0 otherwise).

**Algorithm 1. Testing the existence of community structure**

**Input:** Edge-weight matrix ***W***, confidence intervals *CI*_*max*_, *CI*_*min*_, $CI_{max}^{\prime }$ and $CI_{min}^{\prime }$ at level *α*/4.

*s* ← 0

s←s+I (max. eigenvalue of *T*(***W***) ∈ *CI*_*max*_)

s←s+I (min. eigenvalues of *T*(***W***) ∈ *CI*_*min*_)

s←s+I (max. eigenvalue of *T*_*e*_(*S*(***W***)) ∈ $CI_{max}^{\prime }$)

s←s+I (min. eigenvalue of *T*_*e*_(*S*(***W***)) ∈ $CI_{min}^{\prime }$)

**if** *s* = 4 **then**

 Accept *H*_0_

**else**

 Reject *H*_0_

**end if**

## Simulation study on synthetic data

In this section, we report on a simulation study to evaluate the performance of our method. First, we investigate the validity of using *F*_1_(*x*) in Eq (7) to approximate the distribution of the maximum eigenvalue λ_*max*_ when *n* is finite. Second, we investigate the power of our method when the null hypothesis *H*_0_ is not true.

Third, we compare the performance of our method outlined in Algorithm 1 with other methods. Basically, existing methods consist of two steps. In the first step, a clustering solution for a given graph is produced by a (arbitrary) clustering method. The resulting solution is subsequently compared with clustering solutions for randomized graphs, and is further evaluated with a specific statistic. In this study, we adapt one of the state-of-the-art methods based on clustering entropy (‘CE’, originally designed for a unweighted graph) [14]: $S=-\frac{1}{L}\sum_{\left(i,j\right)}\{p_{i,j}{\text{log}}_{2}p_{i,j}+(1-p_{i,j}){\text{log}}_{2}(1-p_{i,j})\}$ where *L* is the total number of edges in the graph, and *p*_*i*,*j*_ is ‘in-cluster probability’ that measures the proportion of concordance of cluster memberships of nodes *i* and *j* between the given graph and the randomized graph over a number of different noisy contaminations (we set the number of such contaminations to 100). Regarding clustering, to the best of our knowledge, there is no clustering method that is specifically designed to detect community structure based on differences of distribution patterns. As a bail-out procedure, we consider one of the state-of-the-art methods for signed networks: Signed spectral clustering based on a normalized, signed Laplacian method (‘SignedSpec’), which is designed to detect weakly balanced structure of graphs, i.e., positive weights within clusters and negative weights between clusters [6]. We also consider conventional spectral clustering (normalized Laplacian method, ‘ConvSpec’), which is applicable to graphs with positive weights. To apply the method ‘ConvSpec’ in our context, we transform an edge-weight matrix into a positively-weighted matrix by subtracting $\underset{i,j}{min}\;w_{i,j}$ from each weight. Note that the method ‘ConvSpec’ is equivalent to the method ‘SignedSpec’ when edge weights are all positive.

### Data generation

For the data structure in this simulation study, we adopted that in [34], setting the number of clusters to five and cluster size to (10*s*, 20*s*, 30*s*, 40*s*, 50*s*), where we manipulated integer *s*. In this setting, we have 5 × 5 = 25 cluster blocks. In each cluster block, weights were independently drawn from a Gaussian distribution *N*(*μ*_*k*,*k*′_, *σ*_*k*,*k*′_) where *μ*_*k*,*k*′_ and $\sigma_{k,k^{\prime }}^{2}$ are the mean and the variance for a cluster block (*k*, *k*′). We generated 100 datasets for each setting.

### Results

**Fig 4:**
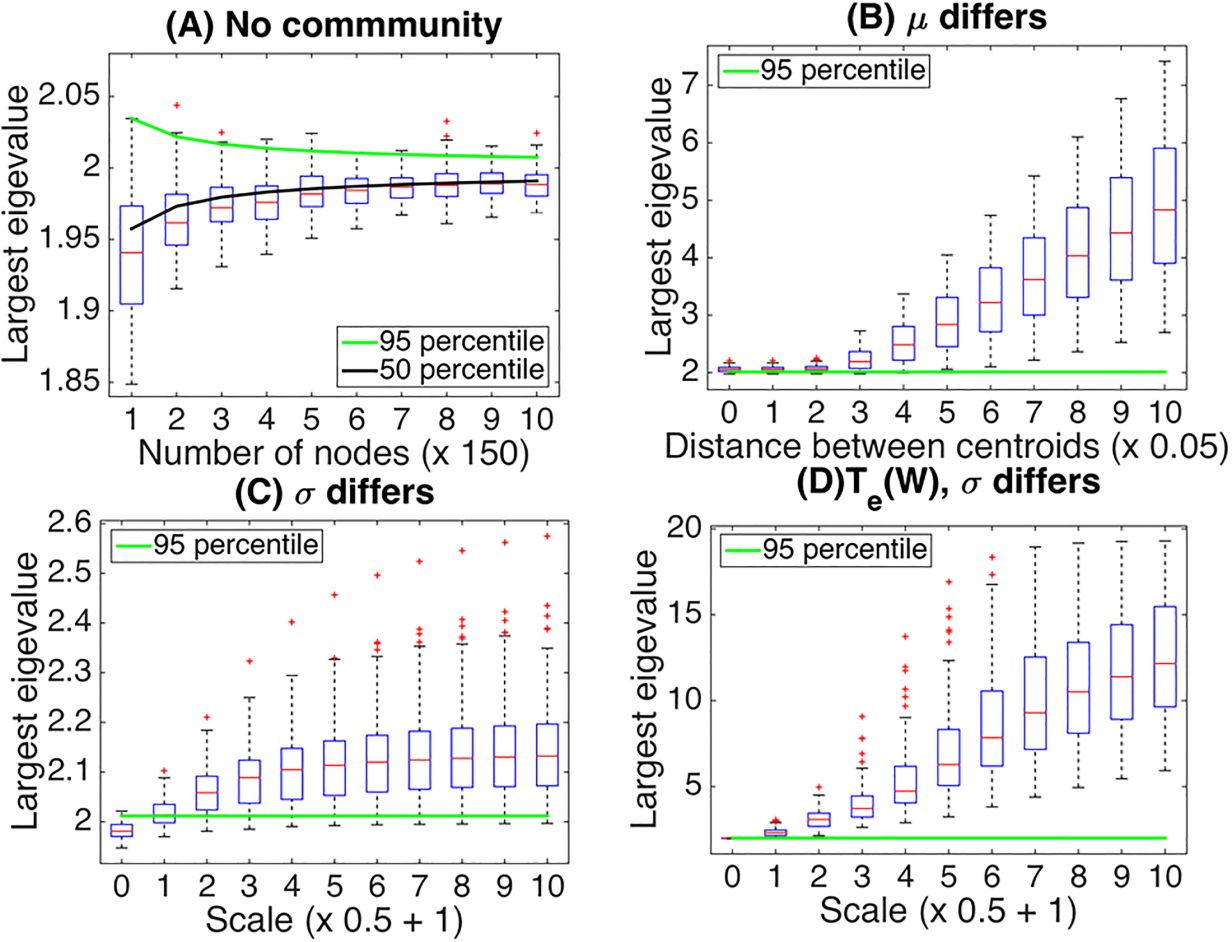
Boxplots represent distributions of the largest eigenvalues for various settings. Panel (A): No-community case (*K* = 1) of Gaussian ensembles for different number of nodes from 150 to 1500 in x-axis. Panel (B): Five-way community case with the number of nodes 750 and cluster size (50, 100, 150, 200, 250). Each cluster block is characterized by means of a Gaussian distribution (while fixing variance = 1), which is randomly chosen from {−*μ*, *μ*} with equal probabilities. The value of *μ* is manipulated from 0 to 0.5 of width 0.1 in x-axis. Panel (C): A five-way community case characterized by variance (while fixing mean = 0), which is randomly chosen from {1, *σ*^2^} with equal probabilities. The value of *σ* is manipulated from 1 to 6 of width 1 in x-axis. Panel (D): A five-way community case in the same setting as in (C), but each edge-weight matrix is transformed by the exponential mapping *Exp* in Eq (4) with *t*_0_ = 1/2. In all panels, the green line denotes the 95 percentile of the largest eigenvalue under the null hypothesis *H*_0_ in (6).

When the number of nodes ranges from 150 to 1500, the distribution function *F*_1_(*x*) in Eq (7) provides a good approximation of the critical value at a significance level of *α* = 0.05 under the null hypothesis *H*_0_ (Fig 4A). Since the function *F*_1_(*x*) provides the asymptotic probability distribution, this result suggests that the function *F*_1_(*x*) also provides a good approximation of the critical value when the number of nodes exceeds this range. In regard to statistical power, it is implied that our method can readily detect the existence of community structure when means *μ*_*k*,*k*′_ in each block differ by at most 0.3 (3 × 0.05 + 3 × 0.05) when *σ*_*k*,*k*′_ = 1 with the number of nodes being 750 (Fig 4B). On the other hand, the power may not be sufficient when differences among cluster blocks are characterized by variances $\sigma_{k,k^{\prime }}^{2}$ (Fig 4C). However, the application of our method to the exponentially transformed matrix by *Exp* considerably improves the power (Fig 4D). All these results suggest good performance of our method in testing for the existence of community structure in a graph.

**Fig 5:**
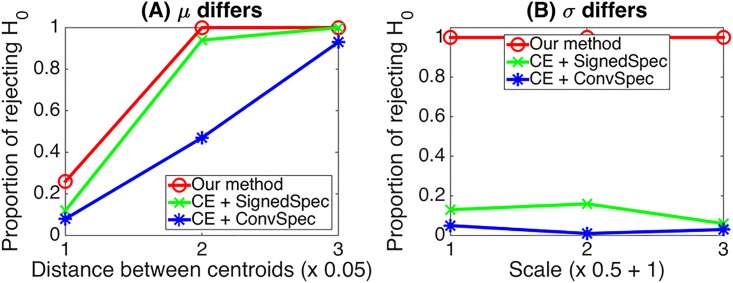
Comparison of the power of the test for three different methods. Our method, the clustering entropy method for the resulting cluster solution using the signed spectral clustering method (CE + SignedSpec), and the clustering entropy method using conventional spectral clustering (CE + ConvSpec). The true community structure is set as follows: cluster size (50, 100, 150, 200, 250); means and variances are manipulated in x-axis of Panel (A) and (B) as in Fig 4B and 4C, respectively.

Lastly, we compare the performance of our method with the remaining methods. We applied our method as outlined in Algorithm 1 to synthetic data, setting *α* to 0.05 (hence, *α*/4 = 0.0125). When the community structure is characterized by mean differences, the performance of our method is comparable with the clustering entropy method with signed spectral clustering (CE + SignedSpec), while it outperforms the clustering entropy method with conventional spectral clustering (CE + ConvSpec) (Fig 5A). On the other hand, when the community structure is characterized by scale differences, our method considerably outperforms other methods (Fig 5B).

## Application to real data 1

In this section, we test our method on real data. The objective is to evaluate the performance of our method when it is applied to various types of real graph data.

### Data

First, we applied our method to the following benchmark graph datasets: Karate club, *Karate* [35]; co-authorships in network science, *Co-authours* [36]; Tribal relationships in highland New Guinea, *Gahuku-Gama* [37]. The datasets of *Karate* and *Co-authours* are binary (i.e., {0, 1}), while the edges in the dataset of *Gahuku-Gama* take discrete signed values, {−1, 0, 1}. The number of nodes for these datasets are 34, 1589, and 16, respectively. These datasets have been well studied in terms of detecting community structure [7].

Second, we applied our method to a real-valued edge-weighted graph: resting state functional MRI (*fMRI*) data [38]. The original dataset consists of the level of BOLD (Blood-Oxygen-Level Dependent) signals at short intervals, which reflects neural activity at tiny regions of the brain, called ‘voxel’ (4949 voxels in this dataset). We pre-processed this dataset by evaluating temporal correlations among these voxels and carrying out Fisher’s z-transformation for them, which results in a 4949 edge-weight matrix ***W***. The objective in this dataset is to test our method on a real-valued, edge-weight matrix and to draw useful inferences from the analysis.

### Results

**Fig 6:**
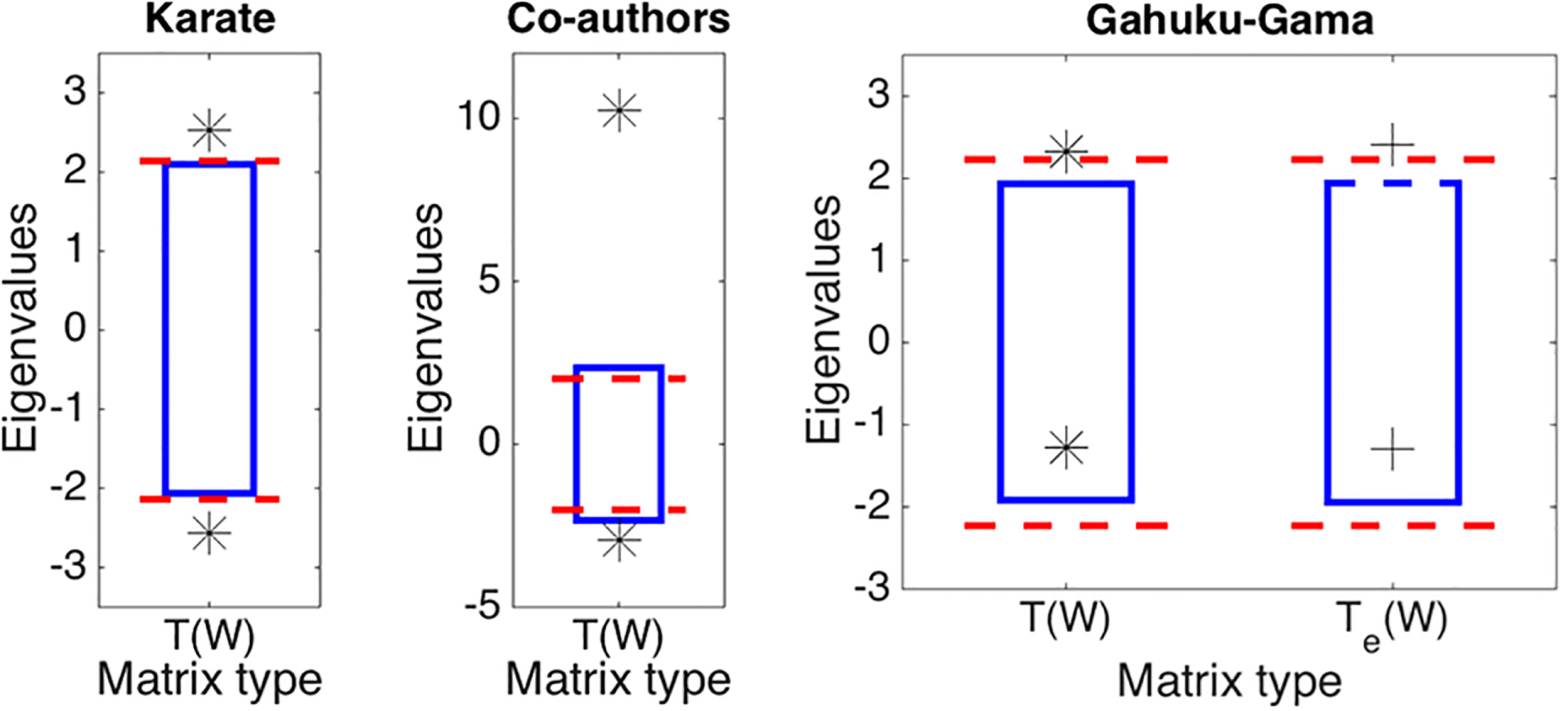
Results of application of our method to real datasets. *Karate*, *Co-authors*, and *Gahuku-Gama* from left to right panels. A star denotes the maximum or minimum eigenvalues of the normalized matrix *T*(***W***), while a cross denotes those of the exponentially normalized matrix *T*_*e*_(*S*(***W***)). The top or bottom edges of boxes denote critical values of these eigenvalues at significance level *α*/2 with *α* = 0.05 for *Karate* and *Co-authors* datasets, and *α*/4 for *Gahuku-Gama* dataset. These critical values resulted from a permutation test with 1000 randomized realizations. In contrast, red dashed lines denote critical values derived from the Tracy-Widom distribution *F*_1_(*x*).

For the first group of real datasets, our method finds some community structure (i.e., *K* > 1), whether we estimate critical values using the Tracy-Widom distribution or a permutation test (Fig 6). Note that in the binary case, we always obtain the same results for the original matrix and for the exponentially transposed matrix, because *T*(***W***) = *T*_*e*_(*S*(***W***)). So, we tested only *T*(***W***) in *Karate* and *Co-authors* datasets, setting the significance level to *α*/2.

It is observed in Fig 6 that confidence intervals largely match between the Tracy-Widom distribution and the permutation test for the *Karate* and *Co-authors* datasets. On the other hand, there is some discrepancy between these for *Gahuku-Gama* data. A possible explanation for this is due to the small number of nodes in the dataset: the Tracy-Widom distribution describes the asymptotic behavior of the eigenvalue when *n* goes to ∞.

**Fig 7:**
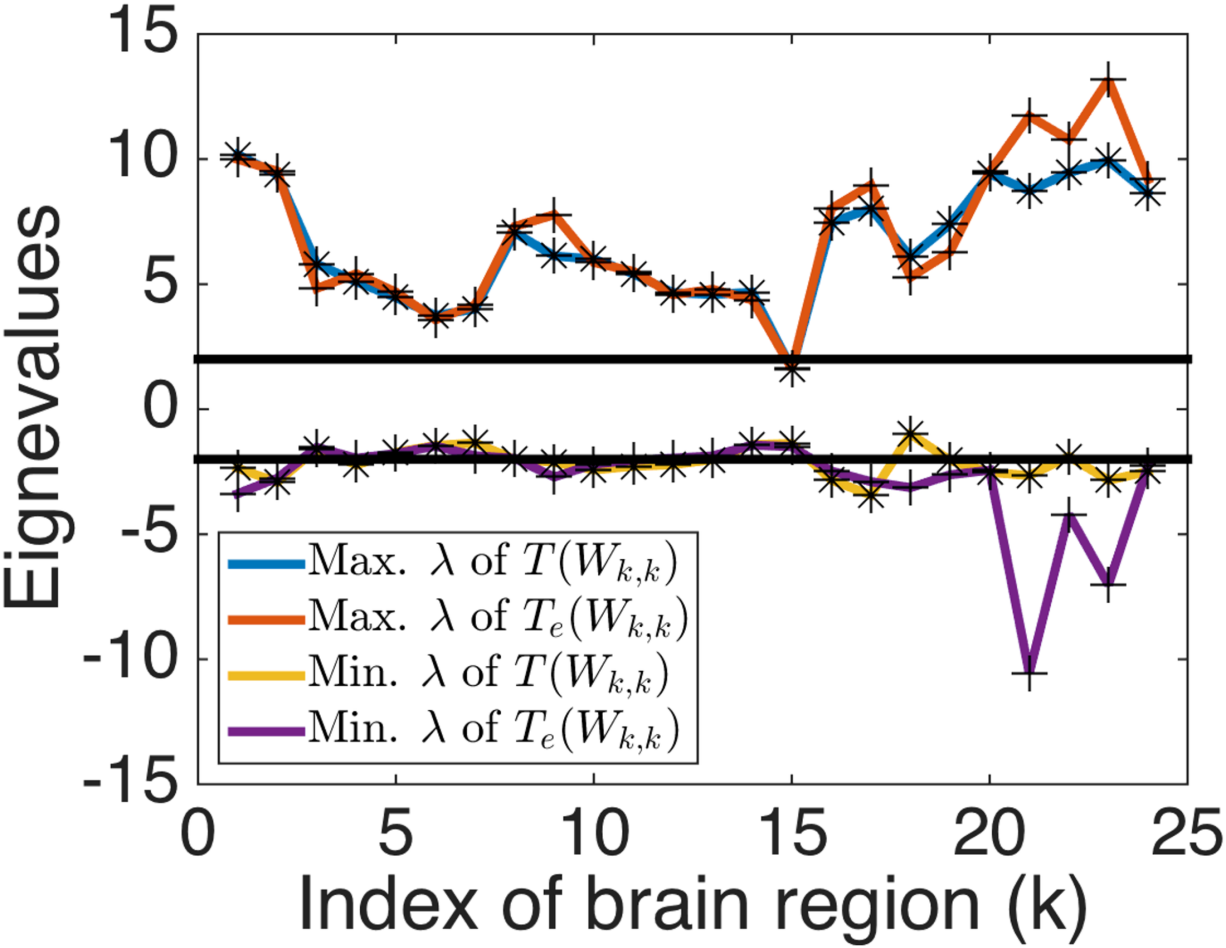
Results of application of our method to the fMRI dataset. Stars denote the maximum or minimum eigenvalues λ for normalized weight matrices by mapping *T* in various brains regions with an edge-weight matrix ***W***_*k*,*k*_, indexed by the brain region *k* in the x-axis. Crosses denote counterparts for exponentially normalized weight matrices by the mapping *T*_*e*_. Horizontal lines denote lines *y* = −2 and *y* = 2, which correspond to values at which the minimum and maximum eigenvalues asymptotically converge.

For *fMRI* dataset, our test rejected the null hypothesis *H*_0_, yielding the maximum and minimum eigenvalues as 31.0 and -7.2 for *T*(***W***), and 31.8 and -10.9 for *T*_*e*_(*S*(***W***)), which provides strong evidence that community structure exists in this graph. Furthermore, we carried out our test for subsets of voxels in brain regions that are anatomically predefined, where the number of voxels ranges from 13 to 498. Our test results suggest that community structure may exist in each region (except for brain region 16) (Fig 7). This result supports the conjecture on heterogeneity of brain activities in anatomically defined brain regions, discussed in the neuroscience literature [39].

## Application to real data 2

We consider further application of our method to real data, focussing in unweighted graphs. The object is to compare its performance and computation time with other relevant methods, which specialize in unweighted graphs. Though our method has been developed for weighted graphs, it works for unweighted graphs as well, because a unweighted graph is a special case of weighted graph. In addition to performance, we also compare computation time in this context.

### Relevant methods

One of the most popular approaches to detection of communities in an unweighted graph is based on ‘modularity’ [40, 41], which is defined as

$$\begin{array}{c} Q=\sum_{k}\left(e_{k,k}-a_{k}^{2}\right), \end{array}$$ (8)

where *e*_*k*,*k*′_ is one-half of the fractions of edges between cluster *k* and *k*′, and *a*_*k*_ = ∑_*k*′_ *e*_*k*,*k*′_. The modularity *Q* denotes deviation of the number of edges from possible random configurations, hence, serving as an objective function for finding a community structure. The algorithm of optimizing *Q* is to start with node-community (a community consisting of a single node) and to aggregate communities to increase *Q* in a similar fashion to a hierarchical clustering algorithm [42]. We use an algorithm of this kind proposed by [41], which is referred to as ‘Newman’. On the other hand, Louvain methods [43–45] are a variant of the modularity-based methods, which optimizes *Q* (or, different type of *Q*) by means of iterating the following two steps. The first step is to optimize *Q* by aggregating communities in the aforementioned manner. The second step is to re-parameterize each community as a single node. These steps are alternatively carried out until no further increment in *Q* is possible. Here, we use one of the most popular methods by [43], referred to as ‘Louvain’. For a threshold of detecting community structure, we use an analytical approximation of modularity for an Erdős-Rényi random graph with *n* nodes and probability *p* of connecting two nodes, which is given as $(1-2/\sqrt{n})(2/(pn){)}^{2/3}$ by [46]. Note that in a sparse graph, even without any community structure, modularity *Q* can take a large value. The analytical approximation captures this point, providing a useful criterion of community detection, though the confidence interval is not readily available. For another relevant method to modularity, we consider an approach based on eigenvalues of a modularity matrix by [47]. In a similar line to the graph Laplacian [48], this method partitions nodes based on the eigenvector of the modularity matrix corresponding to the largest positive eigenvalue. By repeatedly evaluating such an eigenvector, we continue to partition nodes until no further positive eigenvalue is obtained. Here, we use this method (referred to as ‘Split’) for the first partition of nodes, evaluating the largest eigenvalue of the modularity matrix. Furthermore, we consider a versatile approach: a Bayesian clustering method for communities in a graph by [49]. This method explicitly models community memberships as probabilistic parameters, which are optimized in a Bayesian manner (referred to as ‘Bayesian’). Lastly, we include a bootstrap method by [16], which is combined with the community detecting method ‘Newman’, setting the proportion of disturbance to 5% (referred to as ‘Bootstrap’). In this method, for simplicity, we evaluate concordances of community structure between the original graph and bootstrapped graphs by means of Adjusted Rand Index [50] in the same spirit as [51].

For meaningful comparison of computation time among different methods, we ran these methods in the same programming language, Matlab, using publicly available codes for Newman in [52], Louvain in [53] and Bayesian in [54]. For our method, Split method, and Bootstrap method, we ourselves programmed corresponding Matlab codes.

### Data

We consider the following real datasets: Social networks in Indian Villages [55, 56] with 203 nodes and 523 edges (referred to as ‘IndianVillage’); Protein-protein interactions in budding yeast [57] with 2361 nodes and 6600 edges (referred to as ‘Yeast’); Word associations based on empirical studies [58] with 10617 nodes and 63000 edges (referred to as ‘FreeAssoc’; we transformed the original data into an undirected graph by adding edges if there is a connection between nodes in either direction). In addition, we also consider inverted graphs in which the status of an edge is inverted in these datasets (i.e., if there is an edge, it is removed; otherwise, it is added). We expected that this would clarify differences of performance among the methods in question. Finally, we generate weighted versions of these datasets as follows. If there is an edge, a weight is randomly generated from *N*(0, 1), otherwise from *N*(0, 0.01). We apply our method and Bayesian method to these datasets (the remainder of methods are not applicable to a weighted graph).

### Results

For the original datasets, the performance of our method is comparable to other relevant methods, because the existence of community structures is well detected (Table 1). On the other hand, our method suggests the existence of community structures for the inverted graphs as well. Bayesian method and Split method yielded similar results. However, the modularity-based methods Newman and Louvain suggest no community structures while the performance of Bootstrap method is in-between. These differences arise from different (implicit) assumptions in the methods. Our method and Bayesian method focus on differences of patterns in occurrence of edges in communities, while the modularity-based approaches focus only on high density of edges in communists. For practical usage, this implies that one should carefully choose a method, depending on what type of community structure one aims to detect. In case of weighted datasets, both our method and Bayesian method yield similar results on detection of community structures. Lastly, as regards computation time, our method outperforms the remainder of the methods. This is possibly due to that these methods go through a procedure to search for community memberships including the number of communities, while our method does not include such a procedure.

**Table 1:** Results of application to unweighted graphs of real datasets: IndianVillage, Yeast and FreeAssoc. In the column of ‘Type’ in the table, ‘Ori’ denotes the original graph while ‘Inv’ the inverted graph. Further, ‘Ori.w’ denotes the weighted original graph while ‘Inv.w’ denotes the weighted inverted graph. For each cell in the table, computation time and a corresponding statistic to detect community structure are displayed. A star marker in digits denotes that the result supports the existence of community structure. These statistics and critical values are given as follows. For our method, the maximum magnitude of eigenvalues λ is used. The critical value is given by the Tracy-Widom distribution in Eq (7). For Newman and Louvain methods, modularity *Q* is used with the critical value 0.45, 0.48, and 0.29 for IndianVillage, Yeast and FreeAssoc, respectively, based on the analytical approximation of modularity for an Erdős-Rényi random graph. For Split method, a positive largest eigenvalue of modularity matrix λ′ suggests community structure while a negative largest eigenvalue λ′ non-community structure. For Bayesian method, the difference of marginal log-likelihood for *K* = 1 and *K* = 2 (‘Dif’; subtraction of *K* = 1 case from *K* = 2 case) is used. A positive difference suggests community structure while a negative difference non-community structure. For Bootstrap method, we evaluate stability of community structure by means of Adjusted Rand Index (ARI) between the targeted graph and bootstrapped graphs (the number of replicates is set to 100). We compare the median of ARI (mARI) with the distribution of ARI when the targeted graph is randomized. If mARI falls within the 95% confidence interval, it suggests that there is no community structure. Seemingly, this method is not computationally efficient. We were not able to obtain the results for FreeAssoc within 72 hours.

Methods	Type	Datasets		
		IndianVillage	Yeast	FreeAssoc
Our method	Ori	0sec, λ = 3.1*	0sec, λ = 7.8*	3mn, λ = 9.6*
	Inv	0sec, λ = 3.1*	4sec, λ = 7.8*	4mn, λ = 9.6*
Newman	Ori	0sec, *Q* = 0.53*	2mn, *Q* = 0.56*	3hr, *Q* = 0.39*
	Inv	0sec, *Q* = 0.00	2mn, *Q* = 0.00	3hr, *Q* = 0.00
Louvain	Ori	0sec, *Q* = 0.53*	15sec, *Q* = 0.56*	2mn, *Q* = 0.42*
	Inv	1sec, *Q* = 0.00	2mn, *Q* = 0.00	1hr, *Q* = 0.00
Split	Ori	0sec, λ′ = 6.3*	3sec, λ′ = 17.0*	5mn, λ′ = 27.4*
	Inv	0sec, λ′ = 4.5*	3sec, λ′ = 10.1*	6mn, λ′ = 17.0*
Bayesian	Ori	2mn, Dif = 2.3*e*4*	23mn, Dif = 9.4*e*6*	4hr, Dif = 1.8*e*8*
	Inv	2mn, Dif = 4.9*e*3*	15mn, Dif = 8.2*e*6*	4hr, Dif = 1.6*e*8*
Bootstrap	Ori	27sec, mARI = 0.36*	4hr, mARI = 0.50*	> 72hr
	Inv	29sec, mARI = 0.15	6hr, mARI = 0.28*	> 72hr
Our method	Ori.w	0sec, λ = 2.7*	7sec, λ = 2.7*	8mn, λ = 2.5*
	Inv.w	0sec, λ = 2.06*	7sec, λ = 2.00	8mn, λ = 1.99
Bayesian	Ori.w	4mn, Dif = 2.1*e*3*	43mn, Dif = 6.0*e*4*	19hr, Dif = 4.0*e*5*
	Inv.w	4mn, Dif = −3.3*e*1	48mn, Dif = −4.9*e*1	66mn, Dif = −5.8*e*1

## Discussion

We have proposed a novel method for a statistical test for the existence of community structure in an undirected graph that is characterized by the first and the second moments of a generative model for edge weights. This method can be considered a nontrivial extension of the recently proposed method [22] from a binary-valued to a real-valued graph. Unlike the existing methods for real-valued graphs, our method does not need a cluster solution. Hence, we can apply this method even to the nontrivial case of clustering in which edge weights take both positive and negative real values. Also, our approach avoids a nontrivial problem of determining the number of clusters. Further, our method is quite efficient in terms of computation time: We only need to evaluate the eigenvalues of an edge-weight matrix once if we use the Tracy-Widom distribution, which is due to the asymptotic results derived from Random Matrix Theory.

As the next step of analysis, one may wonder how to find community memberships when our test rejects the null hypothesis of *K* = 1. The present paper did not address this issue, but it would be quite useful to examine eigenvectors of the edge-weight matrix as in the case of spectral clustering. It is conjectured that some of the eigenvectors of *T*(***W***) and *T*_*e*_(*S*(***W***)) may contain information on community memberships. In the future, it will be important to determine and to synthesize relevant eigenvectors for inferring underlying community structure.

## Supporting information

S1 Appendix A. Proof of Example 1. (PDF)

S2 Appendix B. Proof of Theorem 1. (PDF)
