# Supplementary material for: Statistical test for detecting community structure in real-valued edge-weighted graphs
Source: PLoS One. 2018 Mar 20;13(3):e0194079. doi: 10.1371/journal.pone.0194079 (PMC5860707; doi:10.1371/journal.pone.0194079)
Supplement: S1 Appendix — (PDF) [file pone.0194079.s001.pdf]

S1 APPENDIX A: Proof of Example 1

The edge-weight matrix can be represented as

$$\boldsymbol{W}_n = \begin{pmatrix} \boldsymbol{W}_{n,1,1} & \dots & \mathbf{0}_{n_1,n_K} \\ \dots & \dots & \dots \\ \mathbf{0}_{n_K,n_1} & \dots & \boldsymbol{W}_{n,K,K} \end{pmatrix},$$

where $\boldsymbol{W}_{n,k,k}$ is the principal submatrix of $\boldsymbol{W}_n$ that consists of cluster block (k,k); n_k the number of nodes in the kth cluster; $\mathbf{0}_{n_k,n_{k'}}$ a $n_k \times n_{k'}$ zero matrix $(1 \leq k, k' \leq K)$. Because of the assumption that $\mu_{k,k'} = 0$, the normalized matrix $T(\boldsymbol{W}_n)$ also has zero off-diagonal blocks. As a result, the eigenvalues of $T(\boldsymbol{W}_n)$ consist of those eigenvalues of cluster block (k,k) in $T(\boldsymbol{W}_n)$. So, it suffices to show that the largest magnitude of eigenvalues of these cluster blocks converge to two (note K is fixed here).

Since the variance $\sigma^2_{k,k}$ for $\boldsymbol{W}_{n,k,k}$ is one, it becomes that the variance of all elements in $\boldsymbol{W}_n$ is $\sum_{k=1}^{K} n_k^2/n^2$. Further, because of the assumption that $n_k = n/K$ $(k = 1, \dots, K)$, the variance of all elements in $\boldsymbol{W}_n$ becomes $1/K$. So, the standardized matrix $S(\boldsymbol{W}_n)$ is given by

$$S(\boldsymbol{W}_n) = \sqrt{K} \times \boldsymbol{W}_n.$$

Hence, the normalized matrix becomes

$$T(\boldsymbol{W}_n) = (\sqrt{K}/\sqrt{n}) \times \boldsymbol{W}_n. \tag{1}$$

Since $n = K \times n_k$, the coefficient $\sqrt{K}/\sqrt{n}$ in Eq.(1) becomes $1/\sqrt{n_k}$. Therefore, the eigenvalues that are relevant for cluster block (k,k) are those eigenvalues of the matrix $\boldsymbol{W}_{n,k,k}/\sqrt{n_k}$. This suggests that the largest magnitude of these eigenvalues converge to two as n goes to ∞.

□
