# Supplementary material for: Statistical test for detecting community structure in real-valued edge-weighted graphs
Source: PLoS One. 2018 Mar 20;13(3):e0194079. doi: 10.1371/journal.pone.0194079 (PMC5860707; doi:10.1371/journal.pone.0194079)
Supplement: S2 Appendix — (PDF) [file pone.0194079.s002.pdf]

S2 APPENDIX B: Proof of Theorem 1

In our setting, if there is no community structure, then obviously, the largest magnitude of eigenvalues of $T(\boldsymbol{W}_n)$ and the eigenvalues of $T_e(\boldsymbol{W}_n)$ converges to two because of universality property of the semicircular law and strong Bai-Yin theorem. Now, we prove the converse. For this purpose, we prove that if there is community structure, then, the largest magnitude of eigenvalues does not converge to two, either for $T(\boldsymbol{W}_n)$ or for $T_e(\boldsymbol{W}_n)$ with some t_0. First, we consider the situation that there is community structure such that some of means $\mu_{k,k'}$ in $S(\boldsymbol{W}_n)$ differ, while variances $\sigma_{k,k'}$ in $S(\boldsymbol{W}_n)$ are the same across different cluster blocks. Note that means and variances are defined for the standardized matrix $S(\boldsymbol{W}_n)$. For simplicity of notation, we denote the normalized weight matrix (i.e., $T(\boldsymbol{W}_n)$) as $\boldsymbol{Z}_n$. The matrix $\boldsymbol{Z}_n$ can be decomposed as follows:

$$\boldsymbol{Z}_n = \boldsymbol{Z}'_n + \boldsymbol{M}_n,$$

where $\boldsymbol{M}_n$ is the normalized mean matrix,

$$\boldsymbol{M}_n = \begin{pmatrix} \boldsymbol{\mu}_{1,1} & \cdots & \boldsymbol{\mu}_{1,K} \\ \cdots & \cdots & \cdots \\ \boldsymbol{\mu}_{K,1} & \cdots & \boldsymbol{\mu}_{K,K} \end{pmatrix} / \sqrt{n},$$

where $\boldsymbol{\mu}_{k,k} = \mu_{k,k}\mathbf{1}_{n_k}\mathbf{1}_{n_k}^T$; n_k the number of nodes in the kth cluster; $\mathbf{1}_m$ a $m \times 1$ vector with elements one. By the dual Weyl inequality [2, p.46] (note that both $\boldsymbol{Z}'_n$ and $\boldsymbol{M}_n$ are symmetric matrices),

$$\lambda_{i+j-n}(\boldsymbol{Z}_n) \geq \lambda_i(\boldsymbol{Z}'_n) + \lambda_j(\boldsymbol{M}_n),$$

where $\lambda_i(\boldsymbol{A})$ denotes the ith eigenvalue of matrix $\boldsymbol{A}$ in descending order. Letting $i = n$ and $j = 1$,

$$\lambda_1(\boldsymbol{Z}_n) \geq \lambda_n(\boldsymbol{Z}'_n) + \lambda_1(\boldsymbol{M}_n). \tag{1}$$

Since elements of $\boldsymbol{Z}'_n$ follow i.i.d. distribution with mean zero and variance one, their eigenvalues are almost surely bounded (between -2 and 2). On the other hand, we evaluate a lower bound of $\lambda_1(\boldsymbol{M}_n)$ as follows. The number of nodes n_k is given by $n_k = r_k \times n$ where r_k is the proportion of the nodes in that cluster. The largest magnitude of eigenvalues is given by the operator norm:

$$\max(|\lambda_1(\boldsymbol{M}_n)|, |\lambda_n(\boldsymbol{M}_n)|) = \sup_{|v|=1} |\boldsymbol{M}_n v|. \tag{2}$$

For simplicity, we assume the left-hand side is given by the largest eigenvalue $\lambda_1(\boldsymbol{M}_n)$ (the following argument is applicable when it is $-\lambda_n(\boldsymbol{M}_n)$ as well). We evaluate a lower bound of $\lambda_1(\boldsymbol{M}_n)$ using Eq.(2). Letting $v = (\boldsymbol{v}_1^T, \ldots, \boldsymbol{v}_K^T)^T$ with $\boldsymbol{v}_k = v_k \times \mathbf{1}_{n_k}$,

$$|\boldsymbol{M}_n v|^2 = \frac{1}{n}\sum_{k=1}^{K} n_k (\sum_{k'=1}^{K} n_{k'}\mu_{k,k'}v_{k'})^2 = n^2 \sum_{k=1}^{K} r_k (\sum_{k'=1}^{K} r_{k'}\mu_{k,k'}v_{k'})^2.$$

Hence,

$$\lambda_1(\boldsymbol{M}_n) \geq \epsilon^2 \times n^2, \tag{3}$$

where $\epsilon^2 = \sum_{k=1}^{K} r_k (\sum_{k'=1}^{K} r_{k'} \mu_{k,k'} v_{k'})^2$. Because of our assumption, there exists non zero $\mu_{k,k'}$ in Eq.(3). This suggests that by appropriately choosing $\boldsymbol{v}$, it becomes that $\epsilon^2 \neq 0$, hence, the largest eigenvalue of $\boldsymbol{Z}_n$ in Eq.(1) takes (infinitely) larger value than 2 as n goes to ∞. Therefore, it is concluded that the largest module of eigenvalues of $\boldsymbol{Z}_n$ does not converge to 2.

So far, we have assumed that the variances of cluster block are the same in $\boldsymbol{Z}$. We relax this condition. Let us assume that variances differ (and means differ). It suffices to show that $\lambda_n(\boldsymbol{Z}'_n)$ in Eq.(1) is lower bounded. We first prove this when $K = 2$. By the dual Weyl inequality,

$$\lambda_n(\boldsymbol{Z}'_n) \geq \lambda_n(\boldsymbol{Z}'_a) + \lambda_n(\boldsymbol{Z}'_b), \tag{4}$$

where

$$\boldsymbol{Z}'_a = \begin{pmatrix} \boldsymbol{Z}'_{11} & \boldsymbol{0} \\ \boldsymbol{0} & \boldsymbol{Z}'_{22} \end{pmatrix}, \ \boldsymbol{Z}'_b = \begin{pmatrix} \boldsymbol{0} & \boldsymbol{Z}'_{12} \\ \boldsymbol{Z}'_{21} & \boldsymbol{0} \end{pmatrix},$$

where $\boldsymbol{Z}'_{i,j}$ is the submatrix of $\boldsymbol{Z}'$ for cluster block (i, j), and $\boldsymbol{Z'}^T_{12} = \boldsymbol{Z}'_{21}$. Obviously, the largest eigenvalues of each diagonal block of $\boldsymbol{Z}'_a$ is bounded, hence, $\lambda_n(\boldsymbol{Z}'_a) = O(1)$. On the other hand, we augment the diagonal blocks for the symmetric matrix $\boldsymbol{Z}'_b$ by generating elements from the same distribution as in its off-diagonal blocks.

$$\boldsymbol{Z}'_{b,arg} = \boldsymbol{Z}'_b + \boldsymbol{Z}'_{b,diag},$$

where $\boldsymbol{Z}'_{b,arg}$ is the augmented matrix and $\boldsymbol{Z}'_{b,diag}$ the diagonal block matrix. Again, by the dual Weyl inequality,

$$\lambda_n(\boldsymbol{Z}'_b) \geq \lambda_n(\boldsymbol{Z}'_{b,arg}) + \lambda_n(-\boldsymbol{Z}'_{b,diag}).$$

Again, the eigenvalues of $\boldsymbol{Z}'_{b,arg}$ and the eigenvalues of $-\boldsymbol{Z}'_{b,diag}$ are bounded. Therefore, it becomes that $\lambda_n(\boldsymbol{Z}'_b)$ is lower bounded. Hence, it is concluded that $\lambda_n(\boldsymbol{Z}'_n)$ is also lower bounded.

Next, we prove that $\lambda_n(\boldsymbol{Z}'_n)$ in Eq.(1) is lower bounded when $K = 3$. In Eq.(4), we consider the following matrices:

$$\boldsymbol{Z}'_a = \begin{pmatrix} \boldsymbol{Z}'_{11} & \boldsymbol{0} & \boldsymbol{0} \\ \boldsymbol{0} & \boldsymbol{Z}'_{22} & \boldsymbol{0} \\ \boldsymbol{0} & \boldsymbol{0} & \boldsymbol{Z}'_{33} \end{pmatrix}, \ \boldsymbol{Z}'_b = \begin{pmatrix} \boldsymbol{0} & \boldsymbol{Z}'_{12} & \boldsymbol{Z}'_{13} \\ \boldsymbol{Z}'_{21} & \boldsymbol{0} & \boldsymbol{Z}'_{23} \\ \boldsymbol{Z}'_{31} & \boldsymbol{Z}'_{32} & \boldsymbol{0} \end{pmatrix}.$$

Obviously, $\lambda_n(\boldsymbol{Z}'_a) = O(1)$. For the matrix $\boldsymbol{Z}'_b$, we decompose it as follows:

$$\boldsymbol{Z}'_b = \begin{pmatrix} \boldsymbol{0} & \boldsymbol{Z}'_{12} & \boldsymbol{0} \\ \boldsymbol{Z}'_{21} & \boldsymbol{0} & \boldsymbol{0} \\ \boldsymbol{0} & \boldsymbol{0} & \boldsymbol{0} \end{pmatrix} + \begin{pmatrix} \boldsymbol{0} & \boldsymbol{0} & \boldsymbol{Z}'_{13} \\ \boldsymbol{0} & \boldsymbol{0} & \boldsymbol{0} \\ \boldsymbol{Z}'_{31} & \boldsymbol{0} & \boldsymbol{0} \end{pmatrix} + \begin{pmatrix} \boldsymbol{0} & \boldsymbol{0} & \boldsymbol{0} \\ \boldsymbol{0} & \boldsymbol{0} & \boldsymbol{Z}'_{23} \\ \boldsymbol{0} & \boldsymbol{Z}'_{32} & \boldsymbol{0} \end{pmatrix}. \tag{5}$$

By the dual Weyl inequality, $\lambda_n(\boldsymbol{Z}'_b)$ is lower bounded by the sum of $\lambda_n(\boldsymbol{Z}_1)$, $\lambda_n(\boldsymbol{Z}_2)$ and $\lambda_n(\boldsymbol{Z}_2)$ where $\boldsymbol{Z}_1$, $\boldsymbol{Z}_2$, and $\boldsymbol{Z}_3$ denote the first, second and third matrices in the right hand side of Eq.(5), respectively. Obviously, eigenvalues of $\boldsymbol{Z}_1$ consist of zeros and eigenvalues of the matrix in which zero blocks from $\boldsymbol{Z}_1$ are removed:

$$\begin{pmatrix} \mathbf{0} & \boldsymbol{Z}'_{12} \\ \boldsymbol{Z}'_{21} & \mathbf{0} \end{pmatrix}. \tag{6}$$

From the reasoning in case of $K = 2$, the eigenvalues of the matrix in Eq.(6) is lower bounded. This suggests that $\lambda_n(\boldsymbol{Z}_1)$ is also lower bounded. In the same manner, $\lambda_n(\boldsymbol{Z}_2)$ and $\lambda_n(\boldsymbol{Z}_3)$ are lower bounded. Hence, it suggests that $\lambda_n(\boldsymbol{Z}'_b)$ is lower bounded. This completes the proof in case of $K = 3$. For case of $K > 3$, the proof is straightforward, because we can always decompose $\boldsymbol{Z}'_b$ as in Eq.(5) where each matrix consists of zeros and homogeneously generated random elements.

Second, we consider the case when means are zero, but some variances differ across cluster blocks. In such a case, we consider the exponential transformation of the edge-weight $\boldsymbol{W}$ where $w_{i,j} \to \exp(t_0 \times w_{i,j})$ (we mention later how to determine t_0). By definition, the expectation of the new variable $\exp(t_0 \times w_{i,j})$ is given by $M_{k,k'}(t_0)$ where $M_{k,k'}(t)$ is the moment-generating function for the distribution of $w_{i,j}$ in block (k, k'). In general, the probability distribution is uniquely determined by the corresponding moment-generating function in an open interval containing zero [1, p.155]. In our current situation, some cluster blocks have different distributions, so, there exists t_0 near zero such that some of $M_{k,k'}(t_0)$ differ. This suggests that some means of the new variables differ. Moreover, if we take t_0 small enough such that $M_{k,k'}(4t_0)$ exists (i.e., at least the fourth moment exists for the exponentially transformed variables; indeed, it is possible to do so, because t_0 can be taken as much small as one wants), strong Bai-Yin theory is applicable to eigenvalues of the new variables. Hence, following the same argument that we have developed in the first case of different means, the largest module of eigenvalues of $T_e(\boldsymbol{W}_n)$ for this specific value of t_0 does not converge to two. This completes the proof that if there is a community structure with zero means, the largest eigenvalue of $T_e(\boldsymbol{W}_n)$ goes to ∞, not converging to two.

□

References

[1] John Rice. *Mathematical statistics and data analysis.* Cengage Learning, 2006.

[2] Terence Tao. *Topics in random matrix theory*, volume 132. American Mathematical Soc., 2012.
